# Interpretation difficulty of normal versus abnormal radiographs using a pediatric example

**Published:** 2016-03-31

**Authors:** Kathy Boutis, Stefan Cano, Martin Pecaric, T. Bram Welch-Horan, Brooke Lampl, Carrie Ruzal-Shapiro, Martin Pusic

**Affiliations:** 1 The Hospital for Sick Children, Toronto; 2 The Peninsula College of Medicine and Dentistry Clinical Neurology Research Group, London UK; 3 Contrail Consulting Services, Toronto; 4 Department of Pediatrics, Texas Children’s Hospital and Baylor College of Medicine, Houston USA; 5 Department of Radiology, Columbia University, New York USA; 6 New York University, New York USA

## Abstract

**Background:**

Radiograph teaching files are usually dominated by abnormal cases, implying that normal radiographs are easier to interpret. Our main objective was to compare the interpretation difficulty of normal versus abnormal radiographs of a set of common pediatric radiographs.

**Methods:**

We developed a 234-item digital case bank of pediatric ankle radiographs, recruited a convenience sample of participants, and presented the cases to each participant who then classified the cases as normal or abnormal. We determined and contrasted the interpretation difficulty of the normal and abnormal x-rays items using Rasch Measurement Theory. We also identified case features that were associated with item difficulty.

**Results:**

139 participants (86 medical students, 7 residents, 29 fellows, 5 emergency physicians, and 3 radiologists) rated a minimum of 50 cases each, which resulted in 16,535 total ratings. Abnormal cases were more difficult (+0.99 logits) than were normal ones (−0.58 logits), difference 1.57 logits (95% CI 1.2, 2.0), but there was considerable overlap in difficulty scores. Patient variables associated with a more difficult normal radiograph included younger patient age (β = −0.16, 95% CI −0.22, −0.10), history of distal fibular tenderness (β = 0.55, 95% CI 0.17, 0.93), and presence of a secondary ossification centre (β = 0.84, 95% CI 0.27, 1.41).

**Conclusions:**

While abnormal images were more difficult to interpret, normal images did show a range of interpretation difficulties. Including a significant proportion of normal cases may be of benefit to learners.

## Introduction

Mastery of radiograph interpretation requires considerable formal training and many hours of practice. While the most common instructional strategy is apprenticeship at the side of an expert practitioner, one of the most time-honored learning aids is the teaching case file. This is typically a collection of images that have been selected by experts for their educational value. More recently, digital technology has facilitated the assembly and use of large teaching files.^1^–^5^

Literature on the diagnostic evaluation of radiograph images can inform the design of educational interventions such as teaching files.^6^–^8^ Norman states that the perceptual components of the radiograph interpretation are only likely to improve from practice with many carefully chosen prototypical examples and variations on the same theme.^9^ From a large number of the examples, the visual system of the learner creates an integrated saliency map such that the salience of a feature on a radiological image is determined partly by the conspicuity of the feature and by prior knowledge and expectations of the learner.^8^ Further, it appears that expert radiologists do not systematically scan a radiographic image but rather detect deviations from a mental representation of the normal image.^7^,^8^ This is the exemplar theory of concept learning where exemplars (e.g. abnormal radiographs) and non-exemplars (e.g. normal radiographs), created within the mind of the learner, are used in future decision making by determining a given case’s similarity to previously encountered exemplars.^10^,^11^ Exemplar-based practice may be more beneficial in early learning resulting in internalized prototypical examples.^12^ If the establishment of exemplar and prototypical examples is a developmental stage on the way to radiological expertise, then establishing a base of “normal” exemplars would be central to that process.

Establishing a base of normal is especially important for front-line physicians since the clinical task is usually to identify pathology in an abundance of normal radiographs. The normal exemplar is especially challenging in pediatric cases where the appearance of a normal film varies with age of the patient and normal variation is common. Nevertheless, most teaching files are heavily skewed towards abnormal examples with relatively few normal cases.^1^–^4^ Thus, the instructional design of current teaching files, with the heavy emphasis on pathologic examples, may be ineffective, since these mostly abnormal file sets do not incorporate the full variability of normal examples (non-exemplars). The implicit and intuitive contrast between normal and abnormal tends to be over-simplified, obscuring the subtle complexities of the compare-contrast exercise which is essential to the learning of concepts.^10^

The proportion of normal compared to abnormal examples seems to make a difference. Teaching files with few normal examples do not simulate a clinical context where it is not known a priori whether an abnormality is present. A higher proportion of normal cases for practice resulted in more missed fractures (false negative rate) with the optimal mix being between 50% and 70% abnormal.^13^ Egglin et al. determined that finding a pulmonary nodule on a given chest radiograph was more difficult when that radiograph was mixed in with a large number of normal radiographs, compared with knowing a priori that there is likely to be a nodule present.^14^ Thus, expectations of those interpreting the radiographs is a contextual factor that influences the likelihood of correctly classifying them.

In this study, we designed a teaching collection that simulates the normal to abnormal ratio and the diagnostic case spectrum of a commonly ordered radiograph in front-line clinical medicine. The task was to classify ankle radiographs taken for the purpose of excluding a fracture into one of two categories: normal or abnormal. We followed the precepts of a) active learning, putting the learner in the position of diagnosing unknown cases in an authentic online environment^15^ and b) deliberate practice by giving feedback with every case and sufficient repetition to allow reinforcement and the creation of internal representations of prototypes.^16^ We then examined the interpretation difficulty of each film based on the readings of participants. To better discern the value of including normal films for a specific trainee level we included learners across a spectrum of medical expertise, from medical students to staff physicians.

Our main objective was to determine the difficulty of classifying normal compared to abnormal radiographs within a set of common pediatric radiographs that reflected a usual clinical practice load. We also examined the learner and patient characteristics that predicted the difficulty of classifying normal radiographs. While we believed that abnormal radiographs would be more difficult to correctly classify and then specify the particular abnormality, we also thought that normal radiograph cases would be difficult for some learners, especially novices. Such a finding would justify including a high proportion of normal cases in teaching files, more than are generally used. Furthermore, we hypothesized that radiographs from younger patients and those with a higher number of ossification centers would be more difficult to interpret accurately.

## Methods

### Study design

This was a prospective, cross-sectional study of medical trainees and faculty that examined the item difficulties of cases in a digital radiology case bank of pediatric ankle radiographs. The cases included both normal and abnormal radiographs.

### Participant recruitment and setting

We recruited a convenience sample of participants: senior medical students, pediatric and emergency medicine residents, fellows and staff in pediatric emergency medicine, and staff radiologists. We solicited participants from three medical schools [Columbia University College of Physicians and Surgeons (New York, USA), University of Toronto Medical School (Toronto, Canada) and Queen’s Medical School (Kingston, Canada)] and postgraduate trainees from two hospitals [The Hospital for Sick Children (Toronto, Canada), and Morgan Stanley Children’s Hospital (New York, USA)]. All participants were approached via electronic mail using directories available from the participating institutions.

### Development of education intervention

#### Radiograph selection

We assembled a set of 234 prospectively collected ankle radiographs in which the indication for the radiograph was exclusion of an ankle fracture.^17^ Pediatric ankle radiographs were chosen because they are a commonly ordered radiograph where we could easily create a set that represented the spectrum of practice for emergency physicians. From our institutional Picture Archiving and Communication System, we downloaded images in JPEG format along with the corresponding final staff pediatric radiology report. For each case, we abstracted a brief clinical history and categorised each case as either normal or abnormal based on the official radiology report. A normal ankle radiograph was defined as a radiograph without a visible bony fracture and/or lack of soft tissue swelling over open growth plates. An abnormal ankle radiograph was defined as a film with a visible bony fracture and/or soft tissue swelling over open growth plates. If there were any uncertainties about the accuracy of the diagnosis on the report it was reviewed with an independent staff pediatric radiologist with specialization in musculoskeletal imaging. Within the 234 radiographs, the diagnoses represented included 131 (56.0%) normal films, 15 (6.4%) normal variants, 76 (32.5%) growth plate fractures, 6 (2.6%) avulsion fractures, 5 (2.1%) cases of a combined tibia/fibula fracture and one demonstrating osteochondritis dissecans. This spectrum is consistent with that seen in a tertiary care pediatric emergency department.^18^

#### Online software application for presentation of radiograph cases

We developed web-based software using HTML, PHP and Flash 8 Professional (Adobe Systems Inc., San Jose, CA) to allow the practice of radiograph interpretation. For each case, the participant initially reviewed a screen listing the presenting complaint and clinical findings. Clicking the appropriate button took the participant to one of the three standard radiographic views of the ankle. The participant could access all three views without time limitation. When ready, the participant diagnosed the case using the following four categories: “Definitely Normal”, “Probably Normal”, “Probably Abnormal” or “Definitely Abnormal.” The Probably/Definitely qualifiers were used to report an index of the participants’ confidence with that item. Further, if the answer was “Abnormal,” the participant was required to mark the radiograph to indicate the location of the abnormality. Participants’ clicking a “Submit” button led to instantaneous feedback. This included a visual overlay indicating the region of abnormality (if any) and presentation of the entire official radiology report. Once the participant considered this information, they moved on to the next case.^17^

### Study administration

Each participant was provided with a unique username and password. Entry to the site was secure. Participants were not informed of the ratio of normal to abnormal radiographs and fracture types included in the exercise, nor were they advised of the intended purpose of the study. They were informed that they had to complete a minimum of 50 cases and would receive feedback after every case, which may result in learning with every case completed. The web application presented the 20 cases that were common to all participants beyond which the participants completed a minimum of 30 more cases presented in a random order specific to each user. All 234 cases were viewed equally frequently. Participants could classify cases at any web-connected computer terminal over as many sessions as they liked. Participants who completed the study were given a $25 gift certificate and had their names entered into a draw to win a prize. The software tracked participant responses, progress through the cases, and time spent reviewing each case; responses were recorded to a mySQL database.

### Outcomes

The primary outcome of interest was the relative interpretation difficulty of the normal compared to abnormal films as determined by a Rasch Measurement Theory (RMT) item analysis.^19^,^20^ We took this approach as RMT offers two primary advantages: 1) the ability to construct linear measurements from ordinal-level data;^21^ and 2) i) item estimates that are free from the sample distribution and ii) person estimates that are free from the scale distribution.^22^ These allow for more sophisticated comparative analyses in situations where different subsets of items are used in a test or scale, as is the case with radiographic interpretations. Secondary advantages included a comparison of the normal to abnormal films with respect to the following variables: 1) patient and radiographic characteristics (age, history of fibular tenderness, effusion, previous fracture, secondary ossification centre, swelling over distal fibula, swelling over distal tibia, normal variant) that may be independently associated with radiograph difficulty for normal ankle radiographs; 2) item difficulties by level of expertise; 3) learner accuracy; 4) learner certainty measured as the proportion of cases reported as “definitely” versus “probably;” 5) time on case measured as time spent interpreting each case reported in seconds from start to end of interpretation; 6) learning curves for normal and abnormal radiographs.

### Data analyses

Each case completed by a participant was considered one item. Normal items were scored dichotomously depending on the match between the participant’s response and the original radiology report (the latter having been determined a priori). Abnormal items were scored correct if the participant had both classified it as abnormal and indicated the correct region of abnormality on at least one of the images of the case.

#### Radiograph difficulty metrics

The main outcome of item (radiograph) difficulty (continuous variable) was determined using the dichotomous simple logistic (Rasch) model.^20^ To ensure that the initial item calibrations were as error free as possible, we checked for differential item functioning by items across subgroups (and found <1%) and report the person-separation index, a measure of reliability (PSI=0.75). We also used linear regression to determine which one of the aforementioned clinical/radiograph variables was independently associated with the dependent variable, Rasch item difficulty. Each candidate variable was regressed against the individual case difficulty as the dependent variable. On multivariate analysis, all variables whose β-coefficient was significant at the 0.10 level were included in the multivariate model. Variables were then dropped if they did not achieve statistical significance to arrive at the final model.

#### Learner metrics

We also examined how case accuracy was dependent on level of expertise, and whether or not the case was normal or abnormal, using a multi-level (cases nested within participants) linear regression model that included the following variables: level of expertise (medical student, postgraduate, or attending), case pathology status (normal or abnormal) and the interaction between the two. Further, we compared the proportion correct (learner accuracy) and the proportion of responses categorized with a diagnostic certainty of “definitely” in the normal versus abnormal radiographs (items) using the Fischer’s exact test. Comparisons of time on case between abnormal and normal items were analyzed with a paired student’s t-test. If the time on case was longer than 5 minutes, we coded that data point as missing since most such observations likely would have been due to the user being interrupted in the middle of a case. The five minute “cut-point” was selected based on a density plot of time-on-case, where 97.4% of participants entered a response within 2.5 minutes. While some were still considering cases for as long as five minutes, after five minutes “time on case” was more likely a representation of a participant leaving the system since time spanned hours to days. Finally, learning curves were plotted for those who completed all 234 cases showing locally weighted scatterplot smoothed curves^23^ (40-case window) of accuracy plotted against number of cases completed.^24^ All analyses were done with SPSS Version 13 (New York, 2004) with the exception of the RMT analyses which were carried out using the Rasch Unidimensional Measurement Models (RUMM) 2030 program.

### Ethics

The research ethics boards at the participating institutions approved this study.

## Results

### Participants

There were 130 participants that rated at least 50 items with 48 completing all 234 resulting in 16,535 ratings in total. Participants included 86 medical students, 7 pediatrics residents, 29 pediatric emergency medicine fellows, 5 staff pediatric emergency physicians, and 3 staff pediatric radiologists. There were 20 (23.3%) medical students, 6 (85.7%) pediatric residents, 12 (41.4%) fellows in pediatric emergency, 5 (100%) staff pediatric emergency and 3 (100%) staff pediatric radiologists that completed all 234 cases.

### Radiograph metrics

#### Rasch item estimates

**Figure 1:**
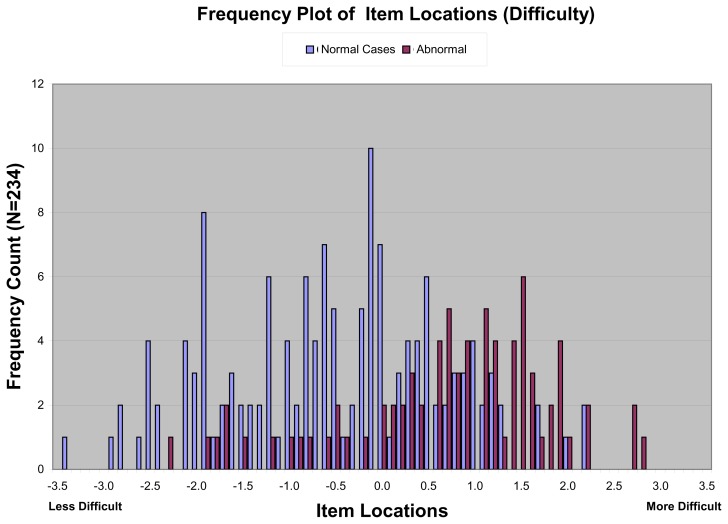
Difficulties of all items as determined by item response theory modeling

Figure 1 (next page) plots the item estimate difficulties on a linear scale derived from RUMM2030, which were distinguished by whether the case showed a fracture or not. There was a complete range of difficulties in both the normal and abnormal radiographs with considerable overlap between the categories. On the Rasch logit scale where mean difficulty has a value of zero with more positive numbers being more difficult, the median (inter-quartile range) difficulty of the normal items (−0.58 logits; −1.48, +0.41) was significantly lower than that for the abnormal items (+0.99; +0.28, +1.61) with a difference of 1.57 logits (95% CI for difference: 1.2, 2.0).

#### Imaging/clinical variables that predict image difficulty

The univariate analysis showed significant associations with normal radiograph difficulty for the following variables: younger age of patient, fibular tenderness on physical examination, distal tibial swelling, distal fibular swelling, and presence of a secondary ossification centre (Table 1). When these variables were entered into the multivariable linear regression model, younger age (β = −0.16, 95% CI −0.22, −0.10; p< 0.0001), history of fibular tenderness (β = 0.55, 95% CI 0.17, 0.93, p=0.005), and presence of a secondary ossification centre (β = 0.84, 95% CI 0.27, 1.41; p=0.004) remained significantly associated with normal radiograph case difficulty.

### Learner metrics

#### Learner accuracy

The mean proportion of cases correct in the normal versus abnormal cases was 76.2% and 52.2%, respectively; mean difference of 24.0% (95% CI 22.6% to 25.4%). As expected, mean accuracy increased with user experience, both overall and when broken out by normal/abnormal status (Table 2). The multi-level regression model showed that significant predictors of accuracy were the three levels of expertise (β=+0.04; 95%CI: 0.03, 0.05 with advanced levels of expertise having higher levels of accuracy), normal versus abnormal status (β= −0.27; −0.30, −0.24 with abnormals being associated with decreased accuracy) and the interaction of expertise and pathology status (β= 0.02; 0.00, 0.23 with expertise having a greater effect on accuracy for abnormals).

#### Learner certainty

Participants chose the qualifier “Definitely” for 30.1% of normal cases, compared with 34.0% for the abnormal cases (95% CI for difference: −9.2%, +16.8%; p=0.5). Thus, participants were no more certain of their answers when presented with a normal or abnormal radiograph.

#### Time on case

The mean (SD) time on case in seconds for normal films versus abnormal was 27.0 (4.0) and 28.0 (6.0), respectively (95% CI for the difference −0.3, 2.3; p=0.1).

#### Learning curves

**Figure 2:**
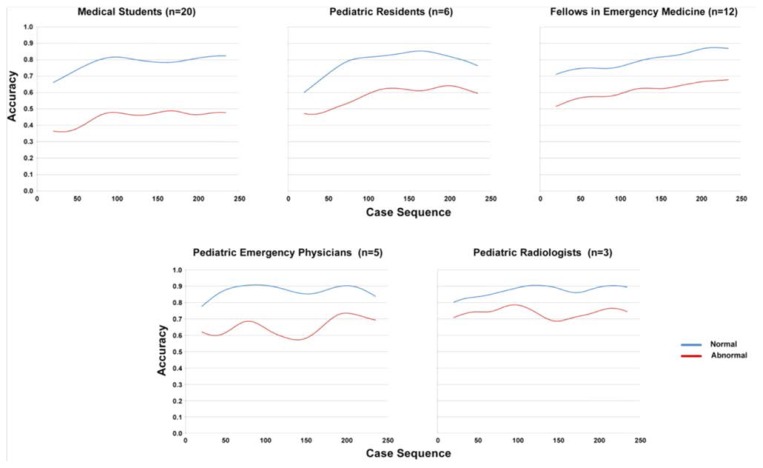
Normal and abnormal case learning curves

A qualitative review of learning curves of normal versus abnormal cases demonstrates that they follow similar patterns (Figure 2).

## Discussion

Using a collection of images in which the ratio of normal to abnormal radiographs was based on actual clinical practice, we demonstrated that the range of interpretation difficulty in the normal cases was comparable to that of abnormal cases. Although abnormal cases were on average more difficult, some normal cases were amongst the most difficult of all. As expected, lower level of learner expertise was associated with a lower level of accuracy identifying normal films. Nevertheless, the learning curves demonstrated knowledge gains from interpreting both normal and abnormal films across the spectrum of expertise. Thus, the interpretation of normal radiographs can be challenging and requires deliberate practice, and these results suggest the inclusion of a higher proportion of normal films in teaching files for at least some content areas and more novice levels of learners.

Novice learners are likely to have limited experience in the range of normal radiographic appearances, to be more susceptible to context effects, and to have more difficulty transferring knowledge to the clinical arena.^11^,^25^–^27^ Thus, a case mix with a higher proportion of normal examples may be appropriate for novice learners. For learners with more expertise who seek efficiency in practice exercises, more cases with pathology may be a better option along with a sprinkling of difficult normal cases. After some expertise is acquired in the more common examples of normal cases, teaching sets could include a smaller number of more difficult normal cases such as radiographs from a younger age cohort and with both clinical tenderness centered around the distal fibula and secondary ossification centers.

Case presentations in teaching files may influence how skills are transferred to real-life clinical settings.^28^,^29^ For example, in most radiograph interpretation teaching experiences, case selection is heavily skewed towards abnormal cases. As a result, the learners focus on finding the abnormality knowing that there is a high probability that it exists. This learning experience stands in stark contrast to most front-line clinical settings where vast majority of radiographs are normal. The educational theory of transfer of learning, where “near” transfer is thought to be easier than “far” transfer, would argue for an approach where the mix of learning cases is nearer to that seen in the clinical context, where a majority are normal.^30^

By taking an RMT approach to this set of radiographs, we have been able to begin the process of better understanding the interpretation difficulty of the spectrum of images encountered by a practicing clinician. The ultimate goal of RMT is to determine the extent to which observed test or scale data satisfy a measurement model, primarily the mathematical embodiment of the principle of invariant comparison.^20^ For this study, we examined the RMT difficulty index and their contribution to concept formation. As a next step, examining the psychometric properties of radiographic images may help us optimize the measurement performance of a set of radiograph cases.^31^,^32^

There are limitations to this study. The results are generated from pediatric ankle radiographs, where normal cases are complicated by developmental variation and the presence of growth plates. Therefore, our results may not generalize to other scenarios where the normal examples are more homogeneous (e.g. adult chest radiographs), and the benefit of a case mix that reflects practice may have less educational value. Our user pool was weighted, by availability, towards the novice end of the spectrum. However, the spread of item “difficulty” between normal and abnormal cases did not narrow with increasing experience. We provided feedback to learners after every case thereby potentially improving learner ability to interpret similar subsequent cases and introducing a confounding variable. This would add noise to our Rasch Item difficulty estimates as would the relatively small number of participants per case. However, the cases were provided to several learners in random order so each case would have raw data with and without feedback. Participants likely varied in attention span, motivation to learn, and how they did the cases (e.g. in one sitting versus over several days). As a result, different participant styles may have affected interpretation scores. However, these variables likely affected performance for normal and abnormal cases equally, and the primary goal of this study was to compare performance on normal vs. abnormal cases, rather than look at the validity of normal/abnormal scores independently. We have not directly demonstrated how an increase in the number of normal cases in teaching files might affect clinical performance and this would be an important area for future study.

In conclusion, while abnormal images were more difficult to interpret, normal images did show a broad range of interpretation difficulties. Thus, including a significant proportion of normal cases may be of benefit to learners, especially more novice learners. Furthermore, as expertise advances, teaching sets could include a smaller set of more difficult normal cases. In our example, this included those from a younger age cohort, clinical tenderness centered on the distal fibula, and radiographs with secondary ossification centers. Future research should explore the educational content and technical aspects of web-based learning to improve the effectiveness and efficiency of physician learning, including the use of computer adaptive learning.

## Figures and Tables

**Table 1:** Univariate analysis of variables potentially associated with normal radiograph difficulty

Variable†	Number of cases	b-coefficient	95% Confidence interval	p-value§
History - age (years)	144	−0.18	(−0.25, −0.12)	<0.001
History - fibular tenderness present	86	0.58	(0.15, 1.01)	0.01
History - tibial tenderness present	29	−0.08	(−0.62, 0.46)	0.76
Radiograph - effusion present	10	0.23	(−0.98, 0.51)	0.54
Radiograph - previous fracture present	5	−0.33	(−0.85, 1.51)	0.58
Secondary ossification centre present	18	1.1	(0.48, 1.73)	<0.001
Distal fibular soft-tissue swelling present	51	0.53	(0.09, 0.98)	0.02
Distal tibial soft-tissue swelling present	24	0.50	(−0.07, 1.08)	0.08
Normal variant present	15	0.43	(0.27, 1.33)	0.23

**Normal radiographs** ***n*** **= 144**

† All predictors are dichotomous (yes/no) except age (continuous in years) and effusion (Small, Medium, Large)

§ Univariate predictors with β-coefficient significant at the p<0.10 level were considered significant and included in the multi-variable regression model.

**Table 2:** Accuracy of participants by level of expertise

	Medical students *n*=86	Residents and fellows *n*=36	Staff physicians† *n*=8	p-value
Normal cases, proportion correct (95% CI)	0.65 (54.90, 75.12)	0.74 (59.67, 88.33)	0.87 (50.53, 81.47)	<0.0001
Abnormal cases, proportion correct (95% CI)	0.45 (34.44, 55.51)	0.55 (38.75, 71.25)	0.68 (52.76, 83.24)	<0.0001
All cases combined, proportion correct (95% CI)	0.57 (46.54, 67.46)	0.66 (50.53, 81.47)	0.80 (66.93, 93.07)	<0.0001

† Includes staff emergency (5) and radiology (3) physicians
